# “Fertile” Mutations in SARS‐CoV‐2 RNA More Frequently Occurred in Hairpin Loops That Determine Virus Evolution

**DOI:** 10.1111/apm.70113

**Published:** 2025-12-21

**Authors:** Philippe Colson, Pierre Pontarotti, Jacques Fantini, Anthony Levasseur, Christian Devaux, Didier Raoult

**Affiliations:** ^1^ IHU Méditerranée Infection Marseille France; ^2^ Microbes Evolution Phylogeny and Infections (MEPHI) Aix‐Marseille Université Marseille France; ^3^ Assistance Publique‐Hôpitaux de Marseille (AP‐HM) Marseille France; ^4^ Department of Biological Sciences Centre National de la Recherche 16 Scientifique (CNRS) Marseille France; ^5^ INSERM UMR UA 16 Aix‐Marseille Université Marseille France; ^6^ Risques Infectieux Tropicaux, Microorganismes Emergents (RITMES) Aix‐Marseille Université Marseille France; ^7^ Aix‐Marseille Université Marseille France

**Keywords:** genome, hairpin, loop, mutation, RNA, SARS‐CoV‐2, secondary structure, stem

## Abstract

RNA hairpins may constitute a foundation of genetic evolution both in viruses and other organisms. Stem‐loops theoretically comprise a stable part, the double‐stranded stem, and a single‐stranded loop allowing evolution. Here we tested for SARS‐CoV‐2 if “fertile” mutations were in loops while mutations in stems were poorly tolerated and rarely found in consensus genomes. We combined information on the frequencies of mutations, either “fertile” (present in ≥ 50 genomes) or “non‐fertile” (neutral, weakly deleterious or lethal) in 61,397 SARS‐CoV‐2 genomes, and on whether these mutations occurred at positions where nucleotides were predicted to be either paired or unpaired. The proportion of positions harboring “fertile” mutations was significantly higher in loops than in stems for the whole genome (11.6% vs. 7.6%; *p* < 0.001, Yates‐corrected chi‐square test). This was also the case in the RNA‐dependent RNA polymerase gene (10.0% vs. 4.9%; *p* = 0.0003) or in the spike gene (12.3% vs. 8.9%; *p* = 0.0049). All four most frequent mutations in our set of genomes were located in loops. Thus, apart from some observations in “accessory” genes, evolution in SARS‐CoV‐2 predominantly occurred in loops while mutations in stems were relatively “non‐fertile.” These stems could be potential antiviral targets, possibly through their disruption by RNA interference.

## 1 Introduction

Virus genomes mutate spontaneously during viral replication, and at various rates [1]. RNA viruses have the highest mutation rate [1]. Mutations that have appeared over time in the genomes of SARS‐CoV‐2, the causative agent of Covid‐19, have generated variants leading to distinct epidemics [2, 3]. Based on a large set of SARS‐CoV‐2 genomes, we have described in previous works that some mutations were associated with “fertility” or even “hyperfertility” as we observed that they were harbored by those genomes with the greatest number of offsprings [3]. In contrast, other mutations were “non‐fertile” as either neutral or weakly deleterious or, for those we called “outlaws,” even associated with the disappearance of the genomes carrying them as they did not appear in the consensus (which we also called “democratic”) genomes [3, 4]. Among the critical elements in SARS‐CoV‐2 evolution, an initial mutation (P323L) in the gene encoding the RNA‐dependent RNA polymerase (RdRp) may have led to a much greater capacity to mutate in the viruses [5]. In addition, another initial mutation (D614G) in the gene encoding the spike allowed a better interaction with the cellular receptor of the virus, ACE2, and increased its infectivity [6].

Secondary structures were described in the SARS‐CoV‐2 RNA genome, which delineated hundreds of stem‐loop hairpins aside large single‐stranded regions, with nearly two‐thirds of the nucleotides being paired [7, 8]. As paired nucleotide regions in RNA genomes were reported to be more stable, and conserved than unpaired regions (either in apical or internal loops or in large single‐stranded regions) [9, 10, 11, 12] and usually under selective pressure to retain the most appropriate conformation for replication [13], we hypothesized that mutations in RNA loops, at positions with unpaired nucleotides, would be more likely to be “fertile” due to their less stable nature compared to stems. Hence, here, we aimed to determine whether previously identified SARS‐CoV‐2 mutations, particularly those located in the RdRp‐ and spike‐encoding genes, and either “fertile” or “non‐fertile,” occurred at genome positions harboring paired or unpaired nucleotides.

## 2 Materials and Methods

We used two different previously published data sets. The first data set consisted of the list of “hyperfertile” (*n* = 220), “fertile” (*n* = 1405), and neutral or weakly deleterious (*n* = 20,600) mutations, classified according to their frequency in the set of 61,397 SARS‐CoV‐2 genomes obtained at our institute between February 2020 and October 2022 [3, 4]. Indeed, these mutations had been detected in more than 835 genomes (high frequency mutations), in between 50 and 835 genomes (medium frequency mutations), and in less than 50 genomes (low frequency mutations), respectively [3]. These thresholds of frequency of mutations had been determined by searching for the most appropriate breakpoints in the distribution of mutation frequencies. In addition, this data set considered 21 strongly deleterious mutations (“outlaws”) [4], which were present in at least one fifth of viral quasispecies characterized for a subset of 90 nasopharyngeal samples, but were completely or almost completely absent among consensus (“democratic”) genomes from our center, or from the GISAID database (https://gisaid.org/) [4, 14]. As a matter of fact, we considered that the most frequent mutations were those beneficial for the viruses whereas those the less frequent were neutral or even deleterious for the viruses. In the present study, due to the low number of “hyperfertile” and “outlaw” mutations, we considered in main analyses only two groups of mutations that consisted of the “(hyper)fertile” mutations (either “hyperfertile” or “fertile” in our previous classification [3]) and “non‐fertile” mutations (either deleterious or, at best, neutral in our previous classification [3, 4]).

The second data set consisted of the list of positions in the SARS‐CoV‐2 genomes where nucleotides were predicted to be either paired or unpaired (as kindly provided by authors from [7]). This information had been obtained in a previous work [7] by determining the SARS‐CoV‐2 RNA genome structure in intact virions using the vRIC‐seq technology, for which SARS‐CoV‐2 virions were trapped on concanavalin A beads by specific interactions with glycoproteins at the virus surface. This technology was developed to analyze small particles such as virions. It was an adaptation of RIC‐seq, an RNA in situ conformation high‐throughput sequencing technology used for unbiased mapping of RNA–RNA spatial interactions in living cells, with a labeling of proximally interacting chimeric RNA with pCp‐Biotin [15].

Present analyses were carried out for all positions of the SARS‐CoV‐2 genome including all its genes. We particularly focused on the RdRp‐ and spike‐encoding genes, on the NSP3 gene that encodes a papain‐like proteinase, on the 5′ untranslated (UTR) region, and on the ORF8 gene that encodes an “accessory” protein. Indeed, the first four genes or regions were those that in our set of genomes harbored the most frequent mutations, which were detected in most or nearly all viral genomes since February 2020. Thus, A23043G, C14408U, C3037U, and C241U were present in 99.8%, 99.7%, 99.7% and 86.4% of the 61,397 genomes from our set, respectively. Regarding the ORF8 gene, it was reported to harbor mutations generating stop codons at 74% of its codons in at least one SARS‐CoV‐2 genome, such mutations being associated in several cases with evolutionary success, and it was reported to be comprised mostly of stem‐loop hairpins [16]. Moreover, some analyses were carried out for categories of SARS‐CoV‐2 genes that had been previously delineated [3] and included (i) structural genes, namely S (spike), E (envelope), M (membrane), and N (nucleocapsid) genes; (ii) “informational” genes (encoding proteins involved in information storage and processing), namely NSP12 (RNA‐dependent RNA polymerase), NSP13 (RNA helicase), NSP14 (3′–5′ exonuclease), NSP15 (endoribonuclease), NSP16 (2′‐O‐methyltransferase), NSP7 and NSP8 (both involved in the replication complex with NSP12), NSP10 (NSP14 and NSP16 cofactor), NSP3, and NSP5 (3C‐like proteinase); (iii) other non‐ structural genes, namely NSP1, NSP2, NSP4, NSP6, and NSP9, which encode proteins with diverse and sometimes imperfectly identified functions; and (iv) “accessory” genes, namely ORF3a, ORF3c, ORF3d, ORF6, ORF7a, ORF7b, ORF8, ORF9b, ORF9c and ORF10, which encode proteins that might modulate viral replication or interfere with host immune responses.

Finally, for statistical analyses, proportions were compared with a Yates‐corrected chi‐squared test or a Fisher exact test with the OpenEpi web application v.3.01 (https://www.openepi.com/); a *p* < 0.05 was considered to be statistically significant.

## 3 Results

**FIGURE 1:**
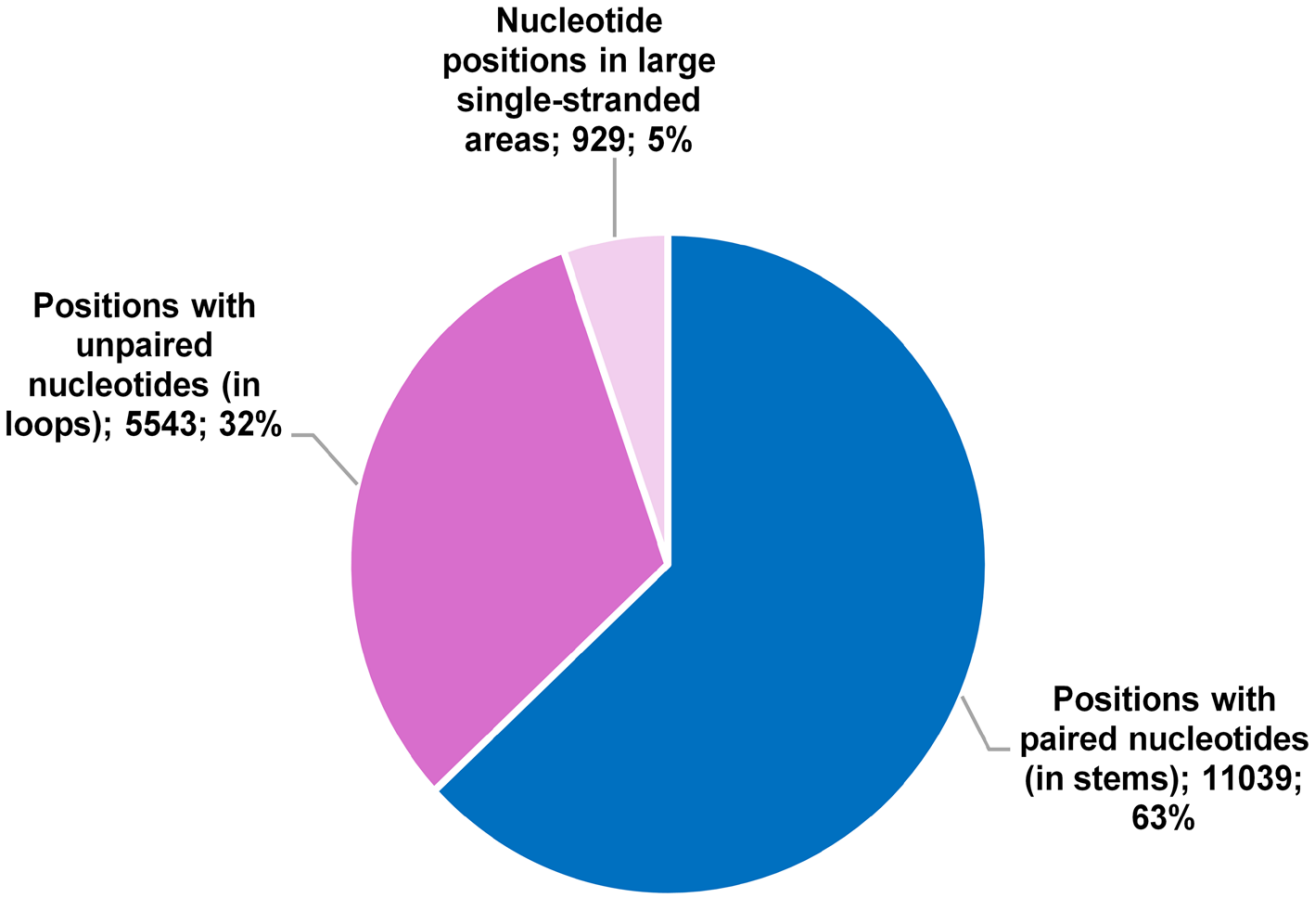
Distribution of positions harboring mutations with paired or unpaired nucleotides in the SARS‐CoV‐2 genome.

**FIGURE 2:**
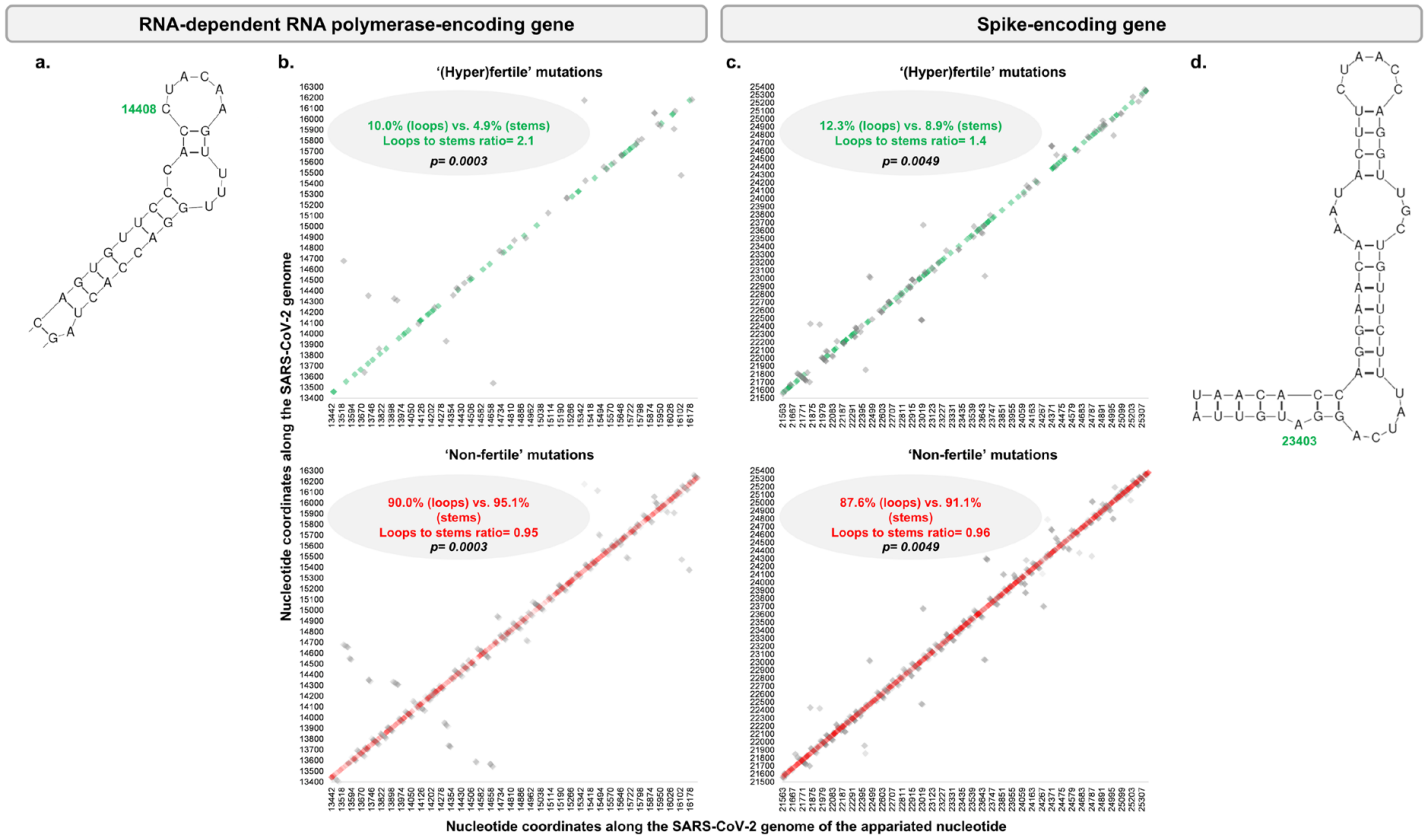
Distribution of nucleotide mutations in loops or large single‐stranded RNA areas and in stems along the RNA‐dependent RNA polymerase and spike‐encoding genes. (a) Secondary structure of a region of the RNA‐dependent RNA polymerase‐encoding genes that harbor the nucleotide position implicated in the critical amino acid substitution P314L; (b) distribution of nucleotide mutations in loops or large single‐stranded RNA areas and in stems along the RNA‐dependent RNA polymerase‐encoding gene; (c) distribution of nucleotide mutations in loops or large single‐stranded RNA areas and in stems along the spike‐encoding gene; (d) secondary structure of a region of the spike‐encoding genes that harbor the nucleotide position implicated in the critical amino acid substitution D614G. In (b) and (c), nucleotide mutations in loops or large single‐stranded RNA areas are as green losanges (indicating “(hyper)fertile” mutations) or as red losanges (indicating “non‐fertile” mutations) while those in stems are as gray losanges. Statistical results are indicated for each comparison (Yates‐corrected chi‐squared test).

At the SARS‐CoV‐2 genome scale, 19,064 (65%) nucleotides were paired, 10,839 (35%) were unpaired among which 9199 were in loops and 1640 were in large single‐stranded areas, according to data from a previous study [7]. Regarding positions that were described to harbor mutations in at least one of the 61,397 genomes obtained in our institute [3], 837 (7.6%) and 10,202 (92.4%) of the 11,039 mutated positions located in stem structures harbored “fertile” and “non‐fertile” mutations, respectively, while 750 (11.6%) and 5722 (88.4%) of the 6472 positions located in loop structures (*n* = 5543) or in large single‐stranded areas (*n* = 929) harbored “fertile” and “non‐fertile” mutations, respectively (Table 1; Figures 1 and 2). For the RdRp gene, 41 (4.9%) and 799 (95.1%) of the 840 mutated positions located in stems harbored “fertile” and “non‐fertile” mutations, respectively, while 47 (10.0%) and 421 (90.0%) of the 468 mutated positions located in loops (*n* = 393) or in large single‐stranded areas (*n* = 75) harbored “fertile” and “non‐fertile” mutations, respectively. For the spike gene, 127 (8.9%) and 1307 (91.1%) of the 1413 mutated positions located in stems harbored “fertile” and “non‐fertile” mutations, respectively, while 105 (12.3%) and 747 (87.7%) of the 852 positions located in loops (*n* = 743) or in large single‐stranded areas (*n* = 109) harbored “fertile” and “non‐fertile” mutations, respectively. For the ORF8 gene, 21 (9.5%) and 200 (90.5%) of the 221 mutated positions located in stems harbored “fertile” and “non‐fertile” mutations, respectively, while 19 (17.1%) and 92 (82.9%) of the 111 mutated positions located in loops (*n* = 102) or in large single‐stranded areas (*n* = 9) harbored “fertile” and “non‐fertile” positions, respectively. Finally, for the 5'UTR, 3 (3.1%) and 93 (96.9%) of the 96 mutated positions located in stems harbored “fertile” and “non‐fertile” mutations, respectively, while 8 (13.8%) and 50 (86.2%) of the 58 positions located in loops (*n* = 50) or in large single‐stranded areas (*n* = 8) harbored “fertile” and “non‐fertile” mutations, respectively.

**TABLE 1:** Frequencies of mutations in 61,397 SARS‐CoV‐2 genomes or quasispecies according to their localization in loops or large single‐stranded areas, or stems of the virus RNA secondary structure.

Genome or gene	Nucleotide size	Mutated positions with paired nucleotides (in stems)									Mutated positions with unpaired nucleotides (in loops or large single‐stranded areas)									Statistics for comparisons of the proportions of positions harboring any “fertile” mutations^1^ in loops or large single‐stranded areas vs. in stems (Yates‐corrected chi‐squared test, except * [Fischer exact test])
		Total nb	Hyperfertile mutations		Fertile mutations		Neutral of weakly deleterious mutations		Outlaw mutations		Total nb	Hyperfertile mutations		Fertile mutations		Neutral of weakly deleterious mutations		Outlaw mutations		
			Nb	%	Nb	%	Nb	%	Nb	%		Nb	%	Nb	%	Nb	%	Nb	%	
Genome	29903	11039	115	1.04	722	6.54	10192	92.33	10	0.09	6472	98	1.51	652	10.07	5711	88.24	11	0.17	**< 0.0000001**
5'UTR	265	96	1	1.04	2	2.08	93	96.88	0	0.00	58	2	3.45	6	10.34	50	86.21	0	0.00	**0.01508** *
NSP1	540	297	2	0.67	20	6.73	275	92.59	0	0.00	127	1	0.79	14	11.02	112	88.19	0	0.00	0.09975
NSP2	1914	860	7	0.81	54	6.28	799	92.91	0	0.00	459	2	0.44	43	9.37	413	89.98	1	0.22	0.05276
NSP3	5835	2194	12	0.55	115	5.24	2066	94.17	1	0.05	1265	15	1.19	129	10.20	1121	88.62	0	0.00	**< 0.0000001**
NSP4	1500	484	5	1.03	25	5.17	454	93.80	0	0.00	296	6	2.03	25	8.45	265	89.53	0	0.00	**0.02168**
NSP5	918	273	3	1.10	19	6.96	251	91.94	0	0.00	164	0	0.00	15	9.15	149	90.85	0	0.00	0.4137
NSP6	870	284	3	1.06	17	5.99	264	92.96	0	0.00	159	6	3.77	14	8.81	139	87.42	0	0.00	**0.03775**
NSP7	249	74	0	0.00	5	6.76	69	93.24	0	0.00	52	1	1.92	3	5.77	48	92.31	0	0.00	0.4402*
NSP8	594	186	2	1.08	5	2.69	179	96.24	0	0.00	116	0	0.00	7	6.03	109	93.97	0	0.00	0.2638
NSP9	339	99	0	0.00	8	8.08	91	91.92	0	0.00	67	1	1.49	8	11.94	58	86.57	0	0.00	0.1963
NSP10	417	126	0	0.00	5	3.97	121	96.03	0	0.00	78	1	1.28	4	5.13	73	93.59	0	0.00	0.3259*
NSP11	39	7	0	0.00	0	0.00	7	100.00	0	0.00	11	0	0.00	2	18.18	9	81.82	0	0.00	0.3595*
NSP12	2795	840	6	0.71	35	4.17	796	94.76	3	0.36	468	7	1.50	40	8.55	416	88.89	5	1.07	**0.0002730**
NSP13	1803	570	3	0.53	39	6.84	528	92.63	0	0.00	318	3	0.94	35	11.01	280	88.05	0	0.00	**0.01524**
NSP14	1581	504	2	0.40	33	6.55	469	93.06	0	0.00	306	3	0.98	25	8.17	278	90.85	0	0.00	0.1595
NSP15	1038	367	0	0.00	22	5.99	345	94.01	0	0.00	218	2	0.92	17	7.80	199	91.28	0	0.00	0.1409
NSP16	894	241	0	0.00	20	8.30	221	91.70	0	0.00	165	0	0.00	15	9.09	150	90.91	0	0.00	0.4604
S	3822	1434	27	1.88	100	6.97	1304	90.93	3	0.21	852	30	3.52	75	8.80	746	87.56	1	0.12	**0.004896**
ORF3a	828	441	5	1.13	48	10.88	388	87.98	0	0.00	277	4	1.44	39	14.08	234	84.48	0	0.00	0.1094
ORF3c	123	83	2	2.41	13	15.66	68	81.93	0	0.00	46	1	2.17	8	17.39	37	80.43	0	0.00	0.4890
ORF3d	171	104	2	1.92	15	14.42	87	83.65	0	0.00	109	2	1.83	19	17.43	88	80.73	0	0.00	0.3529
E	228	81	0	0.00	4	4.94	77	95.06	0	0.00	30	1	3.33	3	10.00	26	86.67	0	0.00	0.1355*
M	669	232	6	2.59	14	6.03	212	91.38	0	0.00	139	3	2.16	15	10.79	121	87.05	0	0.00	0.1246
ORF6	186	90	4	4.44	1	1.11	85	94.44	0	0.00	63	0	0.00	5	7.94	58	92.06	0	0.00	0.3941*
ORF7a	366	218	3	1.38	22	10.09	193	88.53	0	0.00	144	1	0.69	20	13.89	123	85.42	0	0.00	0.2389
ORF7b	132	77	2	2.60	7	9.09	68	88.31	0	0.00	33	0	0.00	2	6.06	31	93.94	0	0.00	0.3004*
ORF8	366	221	6	2.71	15	6.79	200	90.50	0	0.00	111	1	0.90	18	16.22	92	82.88	0	0.00	**0.03347**
N	1260	540	14	2.59	60	11.11	463	85.74	3	0.56	379	3	0.79	50	13.19	321	84.70	5	1.32	0.4903
ORF9b	291	149	6	4.03	13	8.72	130	87.25	0	0.00	71	2	2.82	12	16.90	57	80.28	0	0.00	0.1253
ORF9c	219	115	4	3.48	13	11.30	97	84.35	1	0.87	71	0	0.00	10	14.08	59	83.10	2	2.82	0.4670
ORF10	117	187	3	1.60	18	9.63	166	88.77	0	0.00	131	2	1.53	20	15.27	109	83.21	0	0.00	0.1037
3'UTR	170	158	2	1.27	24	15.19	132	83.54	0	0.00	102	3	2.94	23	22.55	76	74.51	0	0.00	0.05269

*Note:* Statistically significant results (*p* < 0.05) are indicated by a bold font.

Abbreviations: Nb, number; N.c., not calculable; NSP, nonstructural protein; ORF, open reading frame; UTR, untranslated region.

* Statistics calculated using the Fischer exact test.

^1^ Any “fertile” mutations included “hyperfertile” and “fertile” mutations.

When considering genes by categories, for the “informational” genes, 326 (6.1%) and 5049 (93.9%) of the 5375 mutated positions located in stems harbored “fertile” and “non‐fertile” mutations, respectively, while 322 (10.2%) and 2828 (89.8%) of the 3150 mutated positions located in loops (*n* = 2706) or in large single‐stranded areas (*n* = 444) harbored “fertile” and “non‐fertile” mutations, respectively. For the structural genes, 225 (9.8%) and 2062 (90.2%) of the 2287 mutated positions located in stems harbored “fertile” and “non‐fertile” mutations, respectively, while 180 (12.8%) and 1220 (87.1%) of the 1400 positions located in loops (*n* = 1192) or in large single‐stranded areas (*n* = 208) harbored “fertile” and “non‐fertile” mutations, respectively. For the other non‐structural genes, other than “informational” or “accessory” genes, 141 (7.0%) and 1883 (93.0%) of the 2024 mutated positions located in stems harbored “fertile” and “non‐fertile” mutations, respectively, while 120 (10.8%) and 988 (89.2%) of the 1108 mutated positions located in loops (*n* = 949) or in large single‐stranded areas (*n* = 159) harbored “fertile” and “non‐fertile” mutations, respectively. Finally, for the “accessory” genes, 170 (11.4%) and 1328 (88.6%) of the 1498 mutated positions located in stems harbored “fertile” and “non‐fertile” mutations, respectively, while 136 (15.1%) and 765 (84.9%) of the 901 positions located in loops (*n* = 641) or in large single‐stranded areas (*n* = 260) harbored “fertile” and “non‐fertile” mutations, respectively.

**FIGURE 3:**
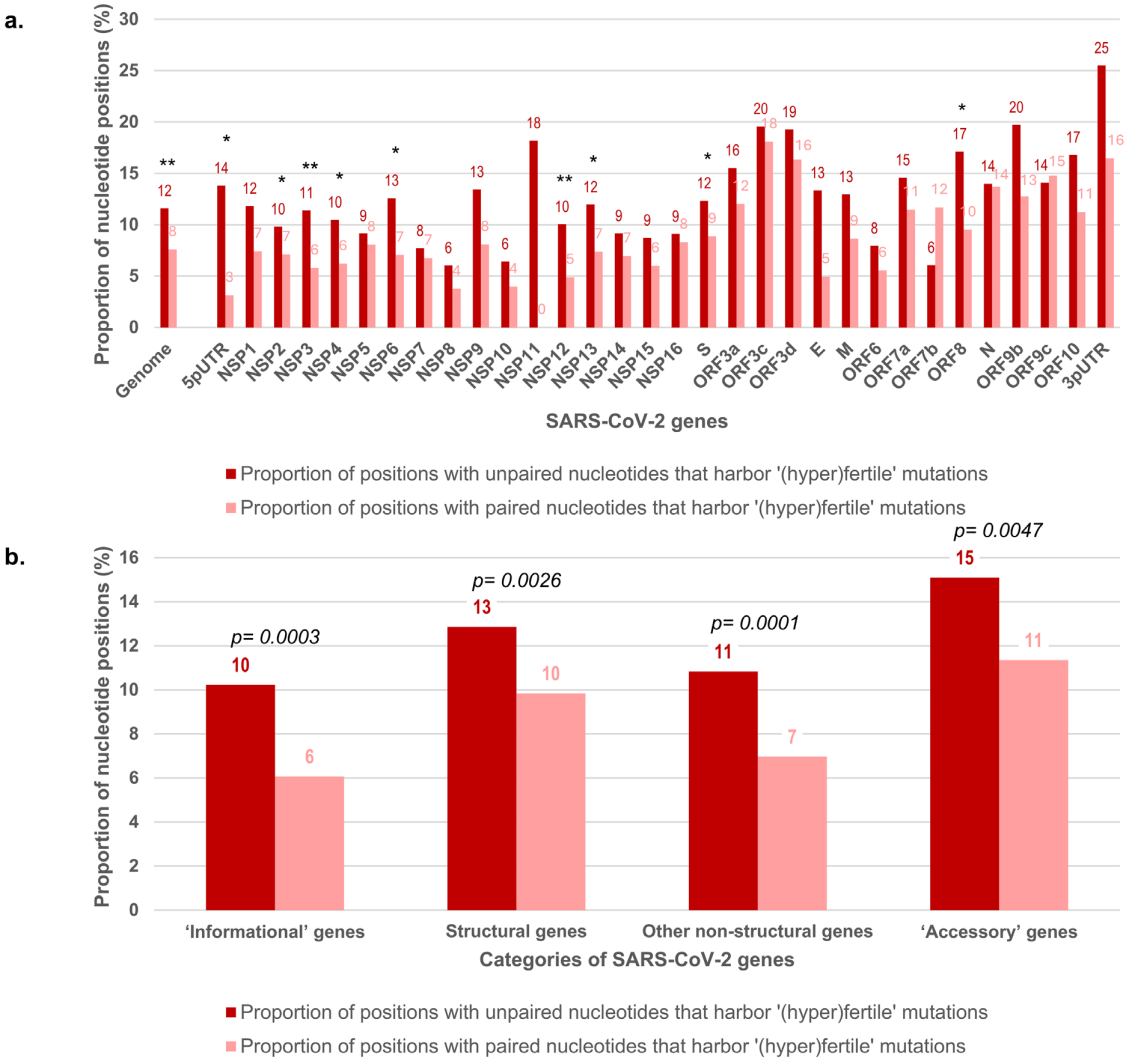
Proportions of nucleotide positions in SARS‐CoV‐2 genomes with unpaired or paired nucleotides that harbor “(hyper)fertile” mutations according to genes (a) or gene categories (b). Statistical data are shown (Yates‐corrected chi‐squared test). (a) **p < 0.001; *p < 0.05.

The proportion of positions harboring “fertile” mutations was significantly higher in loops or large single‐stranded areas than in stems for the whole genome (11.6% vs. 7.6%; *p* < 0.001, Yates‐corrected chi‐squared test), with a loops and single‐stranded areas to stems ratio of 1.53 (Table 1; Figure 3a). This was also the case in the RdRp gene (10.0% vs. 4.9%; *p* = 0.0003) with a loops and single‐stranded areas to stems ratio of 2.1; in the spike gene (12.3% vs. 8.9%; *p* = 0.0049) with a loops and single‐stranded areas to stems ratio of 1.4; in the NSP3 gene (11.4% vs. 5.8%; *p* < 0.001) with a loops and single‐stranded areas to stems ratio of 2.0; in the ORF8 gene (17.1% vs. 9.5%; *p* = 0.0335) with a loops and single‐stranded areas to stems ratio of 1.8; and in the 5'UTR (13.8% vs. 3.1%; *p* = 0.0163, Fisher exact test) with a loops and single‐stranded areas to stems ratio of 4.4. Also, the proportion of positions harboring “fertile” mutations was significantly higher in loops or large single‐stranded areas than in stems for “informational” genes (10.2% vs. 6.1%; *p* < 0.001, Yates‐corrected chi‐squared test) with a loops and single‐stranded areas to stems ratio of 1.7; in structural genes (12.9% vs. 9.8%; *p* = 0.0026) with a loops and single‐stranded areas to stems ratio of 1.3; in other structural genes than “informational” or “accessory” genes (10.8% vs. 7.0%; *p* = 0.0001) with a loops and single‐stranded areas to stems ratio of 1.6; and in “accessory” genes (15.1% vs. 11.4%; *p* = 0.0047) with a loops and single‐stranded areas to stems ratio of 1.3 (Figure 3b). Among SARS‐CoV‐2 genes, ORF7b, which encodes a small membranous accessory membrane protein [17], was an exception with a lower proportion, although not statistically significantly, of positions harboring “fertile” mutations in loops or large single‐stranded areas than in stems (6.1% vs. 9.1%; *p* = 0.3004, Fisher exact test), with a loops and single‐stranded areas to stems ratio of 0.67 (Table 1; Figure 3a).

Regarding positions harboring “hyperfertile” mutations, their number was low, being 213 for the whole genome, and 13, 57, 27, 7, and 3 in the RdRp gene, the spike gene, the NSP3 gene, the ORF8 gene, and the 5′ UTR, respectively. This limited the power of the statistical analyses. Nonetheless, the proportions of positions harboring such mutations were significantly higher in loops or large single‐stranded areas than in stems for the whole genome (1.5% vs. 1.0%; *p* = 0.0037), for the spike gene (3.5% vs. 1.9%; *p* = 0.0110), and for the NSP3 gene (1.2% vs. 0.6%; *p* = 0.0318), while they were more than two times greater for the RdRp gene (1.5% vs. 0.7%) and for the 5′ UTR (3.5% vs. 1.0%), although these differences were not statistically significant (*p* = 0.1419, Yates‐corrected chi‐squared test, and *p* = 0.3175, Fisher exact test, respectively) (Table 1). In addition, the loops and single‐stranded areas to stems ratio was 1.5 overall, and 2.1, 1.9, 2.2, and 3.3 for the RdRp gene, the spike gene, the NSP3 gene, and the 5′ UTR. Moreover, the proportion of positions harboring “hyperfertile” mutations was higher in loops or large single‐stranded areas than in stems for “informational” genes (1.0% vs. 0.5%; *p* = 0.0061, Yates‐corrected chi‐squared test) with a loops and single‐stranded areas to stems ratio of 2.0. It was also higher, although not significantly, in loops or large single‐stranded areas than in stems for structural genes (2.6% vs. 2.1%; *p* = 0.1483) with a loops and single‐stranded areas to stems ratio of 1.3; and for other structural genes than “informational” or “accessory” genes (1.3% vs. 0.9%; *p* = 0.1752) with a loops and single‐stranded areas to stems ratio of 1.4. In contrast, the proportion of positions harboring “hyperfertile” mutations was lower, although not significantly, in loops or large single‐stranded areas than in stems for “accessory” genes (1.3% vs. 2.1%; *p* = 0.1023) with a loops and single‐stranded areas to stems ratio of 0.6.

It is worthy to note that the four mutations A23043G, C14408U, C3037U, and C241U that were the most frequent in our set of 61,397 genomes [3] were all located in loops, as previously highlighted [7]. Moreover, in contrast with what was observed for other genes, for the ORF8 gene, the proportion of positions harboring “hyperfertile” mutations was lower in loops or large single‐stranded areas than in stems with a single (0.9%) position harboring “hyperfertile” mutations among the 111 located in loops or single‐stranded areas compared to 6 positions (2.7%) harboring “hyperfertile” mutations among the 221 located in stems (*p* = 0.2480), the loops and single‐stranded areas to stems ratio being 0.3. As a matter of fact, 6 of the 7 “hyperfertile” mutations were present in stems, while 3 of these 7 “hyperfertile” mutations generated stop codons, and 2 of these 3 latter mutations were located in stems. Interestingly, the same picture was observed for other “accessory” genes, for which 20 (74%) of the 27 “hyperfertile” mutations were present in stems. This was the case for 5 of the 9 “hyperfertile” mutations in ORF3a; for 2 (67%) of the 3 “hyperfertile” mutations in ORF3c; for 3 (75%) of the 4 “hyperfertile” mutations in ORF7a; for the 2 (100%) “hyperfertile” mutations in ORF7b; for 6 (75%) of the 8 “hyperfertile” mutations in ORF9b; for the 4 (100%) “hyperfertile” mutations in ORF9c; and for 3 (60%) of the 5 “hyperfertile” mutations in ORF10. For other genome regions than those corresponding to these “accessory” genes, 95 (52%) of the 184 “hyperfertile” mutations were present in stems; which was a significantly lower proportion than for “accessory” genes (*p* = 0.0239).

Regarding positions harboring “non‐fertile” mutations, they were significantly less frequent in loops or large single‐stranded areas than in stems of the SARS‐CoV‐2 genome, being 92.4% in stems and 88.4% in loops or large single‐stranded areas (*p* ≤ 0.001, Yates‐corrected chi‐squared test), with a loops and single‐stranded areas to stems ratio of 0.96 (Table 1). This was also the case in the RdRp gene, where “non‐fertile” mutations were for 95.1% of the cases in stems and for 90.0% in loops or large single‐stranded areas (*p* = 0.0003; loops and single‐stranded areas to stems ratio = 0.95); in the spike gene where “non‐fertile” mutations were for 91.1% in stems and for 87.6% in loops or large single‐stranded areas (*p* = 0.0049; loops and single‐stranded areas to stems ratio = 0.96); in the NSP3 gene where “non‐fertile” mutations were for 94.2% in stems and for 88.6% in loops or large single‐stranded areas (*p* < 0.001; loops and single‐stranded areas to stems ratio = 0.94); in the 5′UTR where “non‐fertile” mutations were for 96.9% in stems and for 86.2% in loops or large single‐stranded areas (*p* = 0.0151; loops and single‐stranded areas to stems ratio = 0.89); and in the ORF8 gene where “non‐fertile” mutations were for 90.5% in stems and for 82.9% in loops or large single‐stranded areas (*p* = 0.0334; loops and single‐stranded areas to stems ratio = 0.92). Regarding strongly deleterious (“outlaw”) mutations, their number was very low, with 21 in total in the SARS‐CoV‐2 genome, and 8, 4, 1, 0, and 0 in the RdRp gene, the spike gene, the NSP3 gene, the ORF8 gene, and the 5′UTR. Notwithstanding, for these mutations the loops and single‐stranded areas to stems ratio was 0.48 for the RdRp gene and 0.81 for the spike gene, with 3 such mutations located in stems for each of these two genes (38% of those in the RdRp gene and 75% of those in the spike gene). Finally, at the genome level, the proportion of positions in stems was similar among conserved (positions with no mutations observed in our dataset) and mutated positions (64.1% vs. 63.5%, respectively).

## 4 Discussion

The present work shows that “hyperfertile” or “fertile” SARS‐CoV‐2 mutations were more frequently located in loops, formed by the predicted secondary structure of the RNA genome, than in stems. This appears logical since the stem structures formed by the complementary double‐stranded pairing of two distinct areas of the same RNA strand are supposed to be more stable, and conserved in order to protect the sequences that compose them [8, 11, 12, 13]. Loops, in contrast, are unpaired, less stabilized elements, thus being able to be more variable. In the present analysis of thousands of SARS‐CoV‐2 genome sequences obtained by next‐generation sequencing in our institute during the 2020–2022 period, we confirm such a distribution of mutations with a higher frequency in loops than in stems for “hyperfertile” and “fertile” mutations in both the RdRp gene, the spike gene, and in all but one gene or UTR. Interestingly, it was previously noticed that mutations C14408U (P323L) in the RdRp and A23043G (D614G) in the spike (Figure 2a,d), together with two other mutations that occurred very early after the SARS‐CoV‐2 emergence (C241U in the 5′UTR, and C3037U in the NSP3‐encoded proteinase) [3, 6], were located in single‐stranded internal or apical loops [7]. It is striking that these four mutations that were the most frequent in our set of 61,397 genomes were all located in loops [3]. They include two non‐synonymous mutations that had a critical impact on the onset of the SARS‐CoV‐2 pandemics and consisted of substitutions D614G in the gene encoding the spike, which improved interaction with the cellular receptor ACE2 and increased infectivity [6], and P323L in the gene encoding the RdRp that may have led to a greater mutation rate [5]. As a matter of fact, stem‐loop structures including internal RNA base pairing have been well described in various virus RNA genomes including for instance HIV‐1, picornaviruses, GB virus C, hepatitis C virus, dengue virus, SARS‐CoV, or SARS‐CoV‐2, and they have been found to be of considerable importance in the virus biology [7, 8, 18, 19, 20, 21, 22, 23]. In this view, it is worthy to note that RNA structures in viral genomes were reported to modulate viral fitness through effects on RNA stability and packaging, on replication efficacy, and on regulation of transcription or translation [7]. For instance, in flaviviruses, 5′ stem‐loop A and 3′ stem‐loop in untranslated regions (UTRs) were reported to be critical for virus production [24]. In Zika virus, mutations that disrupt RNA–RNA interaction within different regions of the viral genome were described to reduce viral infectivity [25]. In alphaviruses, packaging signals consist of 4 to 6 stem‐loop structures whose mutagenesis strongly decreased viral genome packaging [26]. In HIV‐1, disruption of the structure of the RNA packaging signal in the 5′ leader region of the genome was reported to decrease packaging and infectivity [27]. Nucleotide substitutions located within two 5′ terminal stem‐loops of the human coronavirus 229E RNA genome and inferred to destabilize their structure were predicted to cause major defects in virus RNA synthesis and viral replication [28]. For SARS‐CoV‐2, targeting stem‐loop 1 in the 5′UTR using locked nucleic acid antisense oligonucleotides was described to inhibit virus translation and replication [29].

Our results are congruent with those from a previous study that reported that in the SARS‐CoV‐2 RNA genome, sequence diversity, consisting of multiple represented and evolutionary successful mutations, was lower at positions where nucleotides were predicted to be paired compared with positions where nucleotides were predicted to be unpaired, as determined by the RNAFold tool [8]. This was assessed by analyzing 17,518 SARS‐CoV‐2 genome sequences, and revealed an overrepresentation of unpaired nucleotides at positions exhibiting greater divergence, by approximately two‐fold for the most variable positions. Moreover, in another study, a 6.2 ratio for C → U mutations, which may be signatures of human cellular enzymes of the Apolipoprotein B Editing Complex (APOBEC) family, at unpaired compared to paired nucleotides was reported in the SARS‐CoV‐2 genomes, indicating an almost 12‐fold skew toward unpaired bases at strongly mutated C → U sites [30]. Another study provided congruent data with more frequent C → U mutations at the tips in bulge or loop regions in the viral RNA secondary structure [31].

Interestingly, in the case of “accessory” genes, approximately three quarters of the “hyperfertile” mutations were in stems, which was significantly more than for the other SARS‐CoV‐2 genes. For the ORF8 gene, positions harboring “hyperfertile” mutations were approximately three times more abundant in stems than in loops or large single‐stranded areas. In addition, 2 of the 3 “hyperfertile” mutations that generated stop codons (among all 7 such mutations) were located in stems. Moreover, the ORF7b gene, which was recently reported to encode a small membranous accessory membrane protein [17], was the only SARS‐CoV‐2 gene or region with a higher proportion of positions harboring “fertile” mutations in stems than in loops or large single‐stranded areas. Taken together, these findings may suggest that in the particular case of the “accessory” genes, such as ORF8, disrupting nucleotide pairs in stems might not be deleterious for the virus, and might even be beneficial similarly to what was previously reported for stop codons in ORF8 [16]. Indeed, these “accessory” genes were previously reported to be those where occurred almost all stop codons detected in SARS‐CoV‐2 genomes [3]. In addition, some of these stop codons corresponded to “hyperfertile” mutations, and in the case of ORF8, they were associated with large epidemics with several SARS‐CoV‐2 variants including the Alpha and XBB.1.5 variants [16, 32]. This showed that ORF8 gene inactivation could be associated with an evolutionary success [16]. In a similar way, the disruption of SARS‐CoV‐2 RNA stem structures in “accessory” genes could increase the viral fitness.

A limitation to the present study is that the predictions of the SARS‐CoV‐2 RNA genome that are involved in base‐pairing by the virion RNA in situ conformation sequencing (vRIC seq) technology used by Cao et al. [7], which infers regions of viral RNA genomes involved in base‐pairing using nuclease digestion of portions that are not protected due to base‐pairing then by labeling, ligating and sequencing portions that are involved in base‐pairing, may lack accuracy. Indeed, protection of some regions of RNA genomes from nuclease activity may alternatively rely on interaction with proteins. Therefore, whether or not a SARS‐CoV‐2 RNA genome region is base‐paired or not with another one, and the proportion of regions that are base‐paired in the genome, may be overestimated using the vRIC seq approach. Notwithstanding, in the study by Cao et al. [7], additional analyses were carried out including analyses of evolutionary sequence covariations, of local duplexes as constraints to predict long‐range duplexes, or of thermodynamic likelihood, in order to support that many of the contacts they detected reflected genuine base‐pairing. Beyond, predictions of base‐paired and not base‐paired regions could be improved by choosing RNase with sequence specificity as RNase I that has a preference for single‐stranded RNA [33], while using RNase sequence specificity could be helpful to refine the identification of RNA‐protein binding [34]. Also, gaining a better insight into sites of RNA‐protein interactions by (reverse) RNase footprinting could contribute to increasing the accuracy of RNA secondary structure inference [33, 35]. Additionally, Hidden Markov model‐based (HMM) approaches have been suggested to identify protein‐RNA binding sites [36]. Thus, combining various approaches could enhance the accuracy of the prediction of the viral RNA genome structure.

Notwithstanding, it appeared clear, apart from some observations in “accessory” genes, that the most “fertile” mutations, which were signatures of the different predominant lineages that spread in our geographical area and worldwide, were more frequently located in loops than in stems, and that these loops were therefore the most likely targets leading to changes in viral fitness. Therefore, SARS‐CoV‐2 genome regions in stem conformation and conserved should be functionally significant RNA motifs and could be potential targets for antiviral strategies. These latter could consist for instance of the use of small interfering RNAs, microRNAs, or antisense oligonucleotides to disrupt regions of the overall RNA secondary structure. Such an approach recently used antisense oligonucleotides comprising modified locked nucleic acid bases (LNA) designed to target hairpin stem‐loop RNA elements of Influenza A virus and SARS‐CoV‐2 [37]. The targeted stem‐loop RNA structure of Influenza A virus was reported to mediate in vitro packaging while that of SARS‐CoV‐2 overlaps the 5'UTR and the NSP1 gene. In both cases, LNA targeting the stem regions exhibited an antiviral effect in vitro, and additionally a protective effect on mice from lethal Influenza A virus infections. Further studies could test antiviral strategies targeting these highly conserved RNA structural elements. In addition, they should investigate if such predominant distribution of the most “fertile” mutations in loops of viral genomes is a general phenomenon, which would be facilitated by the availability of large genome sets of various virus species.

## Funding

This study was supported by the French Government under the “Investments for the Future” program managed by the National Agency for Research (ANR) (Méditerranée‐Infection 10‐IAHU‐03). Funding sources played no role in the design and conduct of the study, the collection, management, analysis, and interpretation of the data, and the preparation, review, or approval of the manuscript.

## Ethics Statement

Analyses have been performed using SARS‐CoV‐2 genomes available in sequence databases (see Data Availability Statement). SARS‐CoV‐2 genomes sequenced at our institute and analyzed here had already been described; they had been obtained through genomic surveillance, as recommended by the French government (https://www.santepubliquefrance.fr/dossiers/coronavirus‐covid‐19/consortium‐emergen). Genome sequencing for SARS‐CoV‐2 genomes reported here had already been approved by the Ethical Committee of the Méditerranée Infection institute under reference no. 2022‐041 [4] and the present study has been linked to the Health Data Access Portal of Marseille university and public hospitals (Assistance Publique‐Hôpitaux de Marseille (AP‐HM)) which was registered with no. PADS24‐190 and was approved by the Ethics and Scientific Committee of AP‐HM.

## Conflicts of Interest

D.R. declares grants or contracts and royalties or licenses from Hitachi High‐Technologies Corporation (Tokyo, Japan) until 2023. P.C. is a scientific adviser of BioSellal and Triber companies. C.D. declares a link of interest with the Sanofi and Merck pharmaceutical companies. The other authors have no conflicts of interest to declare relative to this study. Funding sources played no role in the design and performance of the study, the collection, management, analysis and interpretation of the data, or the preparation, review and approval of the manuscript.

## Data Availability

The data that support the findings of this study including the lists and nature of SARS‐CoV‐2 mutations are openly available in GenBank at https://www.ncbi.nlm.nih.gov/genbank/, in GISAID at https://gisaid.org/, and through references [3] and [4].
